# Outcomes of malignant orbital tumors: a 22-year Norwegian single-center series

**DOI:** 10.3389/fsurg.2026.1862307

**Published:** 2026-06-24

**Authors:** Bendik Alvheim Sundfjord, Julie Selvik Bentzon, Signe Johnsen Landa, Svein Arthur Jensen, Dorota Goplen, Jon Espen Dale, Stein Lybak, Hans Olav Ueland, Eyvind Rødahl

**Affiliations:** 1 Department of Clinical Medicine, University of Bergen, Bergen, Norway; 2 Department of Oncology, Haukeland University Hospital, Bergen, Norway; 3 Department of Radiology, Haukeland University Hospital, Bergen, Norway; 4 Department of Plastic, Hand and Reconstructive Surgery, Haukeland University Hospital, Bergen, Norway; 5 Department of Otolaryngology/Head and Neck Surgery, Haukeland University Hospital, Bergen, Norway; 6 Department of Ophthalmology, Haukeland University Hospital, Bergen, Norway

**Keywords:** 5-year survival, exenteration, lymphoproliferative disorders, malignant tumors, metastases, orbital tumors, squamous cell carcinoma, radiotherapy

## Abstract

**Purpose:**

To evaluate outcome data for patients with malignant orbital tumors treated at Haukeland University Hospital over a 22 year period and compare them with existing literature.

**Methods:**

Patients were identified by searching for relevant codes in the Hospital's database. Medical records from patients with malignant orbital tumors treated from 1999 to 2020 were retrieved. Clinical features, management, and outcome was recorded. Incidence was estimated from yearly population data provided by Statistics Norway.

**Results:**

We included 76 patients, 41 (54%) females and 35 (46%) males corresponding to a yearly incidence in Western Norway of 0.30 per 100 000 inhabitants. Median age at time of diagnosis was 64 years (3-97 years). Secondary invading tumors were most frequently seen (46%), followed by lymphoproliferative (24%), metastatic (16%) and primary orbital tumors (14%). Biopsy and histopathologic confirmation were obtained in 73 out of 76 patients (96%). Of 40 different histological subgroups, squamous cell carcinoma from paranasal sinuses was the most common (16%). A total of 42 patients (55%) had their tumor surgically removed, of which 23 patients (55%) underwent exenteration. Local radiotherapy was administrated to 55 (72%) patients and 29 (38%) patients received chemotherapy. One or more local/regional relapses or distant metastases were seen in 32 (42%) patients. Overall disease specific 5-year survival was 59%, with significant differences between subgroups; lymphoproliferative diseases 82%, primary orbital tumors 80%, secondary invading tumors 46%, and orbital metastases 39%.

**Conclusion:**

The spectrum of malignant orbital tumors, treatment modalities, and survival rates aligned closely with international literature. While our findings demonstrate that high-quality care is feasible at a low-volume center, further studies on the volume-outcome relationship in orbital oncology is warranted.

## Introduction

Orbital malignancies are rare with an estimated yearly incidence of approximately 1 in 300 000 individuals. Reported rates of malignancy among orbital tumors vary between 29% and 63% in different series (1–8). The condition can occur in individuals of all ages with a peak incidence between the sixth and seventh decades of life (3, 9). Globally, the incidence of orbital malignancies appears to be increasing, largely due to a higher occurrence of orbital lymphomas (5–7, 9–11).

Orbital malignancies may occur anywhere in the orbit, but most of them are located in the extraconal space (2). Primary tumors originate from the orbital tissues. While some investigators categorise lymphoproliferative disorders as a primary orbital tumor, others consider them as a separate entity. Secondary tumors include tumors invading from adjacent structures like paranasal sinuses, nasal cavity, lacrimal sac, eyelids, conjunctiva, eyeball, or surrounding skin, and distant metastases.

Guidelines for diagnosis and treatment of certain orbital malignancies have been established (12–14). A variety of therapeutic options exists depending upon histological features and staging of the tumor. Prompt diagnosis and treatment offer the best prognosis (1, 15). During the past decades, there has been a continuous development in diagnostics (16), imaging techniques (17–22), microsurgery (23–26), and multimodal treatments with radiotherapy, chemotherapy, and immunotherapy (27–32) that have led to less invasive treatments with improved outcomes (33).

Treatment of patients with rare disorders can be challenging, particularly for teams that serve relatively small populations. Volume as a predictor of outcome has been a topic of interest for many years (34). For most procedures/diseases including cancers, high-volume hospitals tend to do better than low-volume hospitals (35). No studies have been performed for orbital malignancies but for head and neck cancers, high-volume surgeons and hospitals generally have better outcomes. This applies to survival rates (36), disease free margins (37), postoperative mortality, and treatment of complications (38).

The aim of the present study was to evaluate the spectrum of diagnoses, treatment modalities and survival rates of patients with malignant orbital tumors seen at a low-volume center and compare these against results from similar studies at other institutions.

## Materials and methods

### Study design and ethical approval

The study is a retrospective review of medical records from patients with orbital malignancies seen at the Department of Ophthalmology, Haukeland University Hospital from 1999 to 2020. Haukeland University Hospital (HUH) is a referral centre for orbital disorders serving Western Norway with approximately 1.1 million inhabitants. Due to a long-standing interest in orbital disorders, HUH also receives patients from other parts of the country.

The study was approved by The Regional Committee for Medical and Health Research Ethics, Western Norway (IRB# 00001872), reference number 162145. The patients were identified by searching for relevant codes in the Hospital's database (see Supplementary Information). A written informed consent was obtained from all living patients. The study thus adhered to the tenets of the Declaration of Helsinki.

### Data recorded

The data collected was age, gender, histological diagnosis, time of diagnosis, treatment, time to local recurrence, regional recurrence (includes regional lymph nodes, parotid gland and facial cutaneous metastases), or distant metastasis, and survival time. Staging was performed according to the Ann Arbor (lymphoproliferative diseases) or TNM classification (7th Edition) systems. If patients underwent repeated surgical interventions, they were recorded either with the first surgical procedure (incisional biopsy excluded) at or after diagnosis of orbital involvement or with exenteration if that had been performed at a later stage. The main endpoints were overall survival (OS), disease specific survival (DSS), and time to recurrence (See Supplementary Information for Definitions).

### Statistical analysis

Statistical analyses were performed with IBM SPSS Package, version 29 (IBM Corporation, Armonk, New York, USA). Continuous data was given as median (range) or median (95% CI) and categorical as number (percent). Survival endpoints were presented by Kaplan–Meier plots. Different tumors were compared with log-rank test where *p* < 0.05 was considered statistically significant. Owing to the limited number of patients in each category, statistical analyses were conducted only when considered appropriate. Yearly incidence of malignant orbital tumors in Western Norway (Vestland and Rogaland counties) was estimated from population data from Statistics Norway (ssb.no).

## Results

Out of 82 patients identified, 76 patients were included in the study. Three patients were excluded because of uncertain orbital involvement, while informed consent could not be obtained for three patients. Three patients with orbital metastases from known primary tumors but without histologically verified diagnosis of the orbital presentation were included because of strong clinical and radiological evidence of malignancy. A total of 64 patients were referred from Western Norway, 12 patients came from other parts of the country. We observed a yearly incidence of one malignant tumor per 336 846 inhabitants, or 0,30 tumors per 100 000 inhabitants in the population from Western Norway.

**Figure 1:**
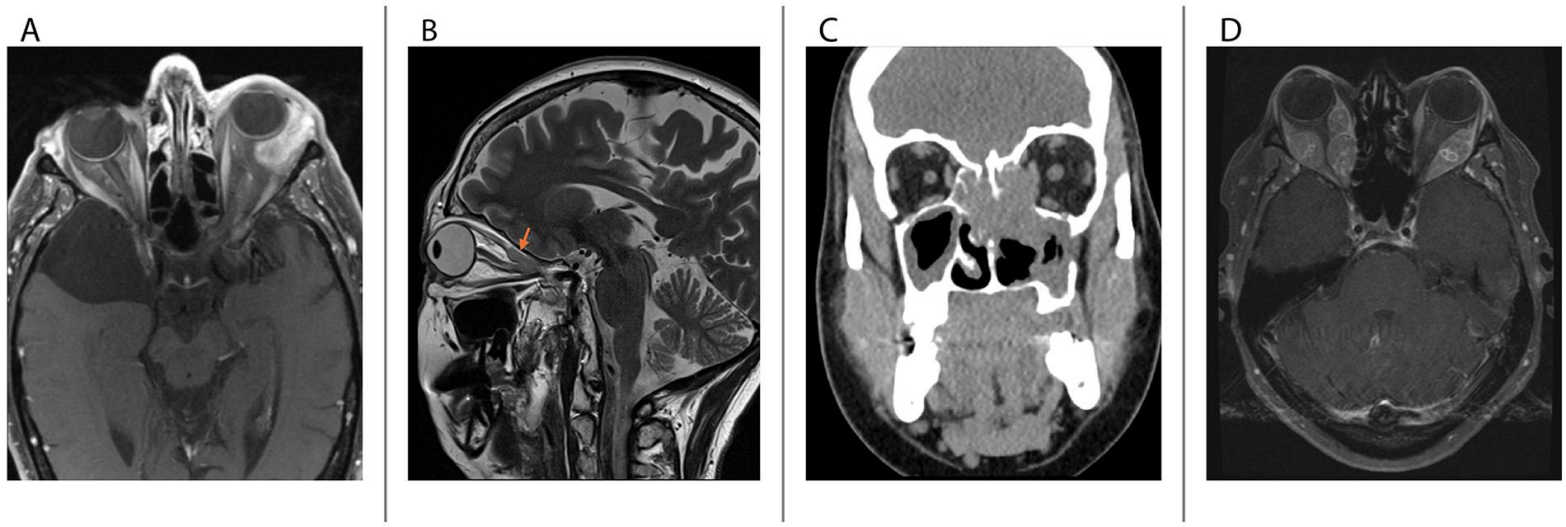
CT and MRI images of primary and secondary orbital tumors. (**A**) Axial T1 weighted MRI with contrast administration of a carcinoma ex pleomorphic adenoma from the left lacrimal gland. (**B**) Sagittal T2 weighted MRI of a diffuse large cell B-cell lymphoma in the apex of the right orbit (arrow). (**C**) Coronal CT scan showing infiltrative growth mainly into the left orbit from a nasal adenocarcioma. (**D**) Axial, fat suppressed, T1 weighted MRI with contrast administration showing multiple metastases to the extraocular muscles in both orbits from a malignant melanoma of the skin.

**Figure 2:**
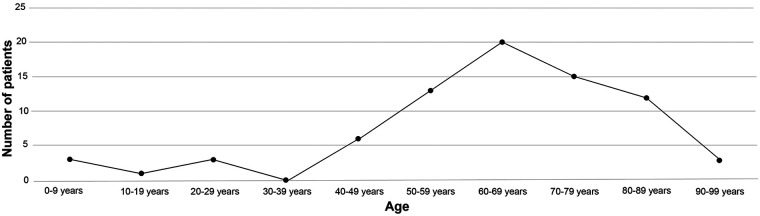
Age range distribution at time of diagnosis.

Radiological imaging with computed tomography (CT) or magnetic resonance imaging (MRI) was performed in 75 out of 76 patients (see Figure 1). Histological diagnoses, gender and age are presented in Table 1 and Figure 2. Involvement was unilateral in 73 patients (96%) and bilateral in three patients (4%). Median duration of symptoms before diagnosis was four months (0–36 months).

**Table 1:** Histopathological classification of 76 patients with malignant orbital tumors at HUH from 1999 to 2020.

Histological subtype and location	Total	Women	Men	Median age, years (range)
**Primary orbital tumors**	11	5 (45%)	6 (55%)	61 (4–81)
Lacrimal gland adenoid cystic carcinoma	3	2	1	68 (64–75)
Lacrimal gland squamous cell carcinoma	1	0	1	54
Lacrimal gland adenocarcinoma	1	0	1	63
Extramedullary myeloid sarcoma	1	1	0	7
Malignant solitary fibrous tumor	1	1	0	61
Carcinoma ex pleomorphic adenoma from lacrimal gland	1	0	1	55
Poorly/undifferentiated carcinoma from lacrimal gland	1	1	0	81
Embryonal rhabdomyosarcoma	1	0	1	4
Undifferentiated pleomorphic sarcoma	1	0	1	53
**Secondary orbital invasion**	35	13 (37%)	22 (63%)	63 (18–97)
Squamous cell carcinoma from paranasal sinuses	12	1	11	67 (46–85)
Squamous cell carcinoma from adjacent skin	4	2	2	73 (66–92)
Sinonasal undifferentiated carcinoma from paranasal sinuses	2	1	1	49–64
Adenocarcinoma from paranasal sinuses	2	0	2	53–83
Conjunctival malignant melanoma	2	2	0	83–97
Adenocarcinoma from lacrimal sac	2	0	2	58–60
Squamous cell carcinoma from lacrimal sac	1	1	0	56
Adenoid cystic carcinoma from lacrimal sac	1	0	1	41
Radiation-induced osteosarcoma from paranasal sinus	1	0	1	18
Adenocarcinoma from nasal cavity	1	0	1	25
Chondrosarcoma from skull base	1	1	0	27
Angiosarcoma from nasal cavity	1	1	0	28
Choroidal malignant melanoma	1	1	0	55
Malignant odontogenic myxoma	1	1	0	63
Nasopharyngeal carcinoma	1	0	1	67
Malignant melanoma from adjacent skin	1	1	0	70
Sebaceous gland carcinoma from eyelid	1	1	0	82
**Lymphoproliferative disorders**	18	16 (89%)	2 (11%)	67 (48–90)
Extra nodal marginal zone lymphoma	7	6	1	60 (48–90)
Follicular lymphoma	4	4	0	69 (53–82)
Diffuse large B-cell lymphoma	4	4	0	78 (62–85)
B-cell lymphoma not otherwise specified	1	1	0	50
Lymphoplasmacytic lymphoma	1	0	1	69
Plasma cell myeloma/solitary plasmacytoma	1	1	0	76
**Metastases (primary tumor location)**	12	7 (58%)	5 (42)	70 (3–87)
Mammary cancer	3	3	0	64 (60–71)
Gastrointestinal tract carcinoid	2	1	1	65–81
Malignant melanoma (1 skin^a^, 1 unknown origin)	2	1	1	47–73
Prostate cancer	1	0	1	86
Fibrosarcoma from sacrum^a^	1	0	1	68
Small bowel leiomyosarcoma	1	1	0	79
Adrenal gland neuroblastoma	1	1	0	3
Cutaneous merkel cell carcinoma^a^	1	0	1	87
Total	76	41 (54%)	35 (46%)	64 (3–97)

a orbital lesion not histologically verified.

Overview of treatment is presented in Tables 2, 3. Surgical procedures (incisional biopsies excluded) were performed in 42 (55%) patients. Exenteration was performed at some point in 23 patients. Twenty-eight patients underwent repeated surgical interventions*.* Radiation therapy (see Supplementary Information for details) was administered in 55 (72%) patients, of which 45 with curative intent, seven with palliative intent, and three with first curative and later palliative intent. Chemotherapy was used in 29 (38%) patients, of which 18 with curative intent, ten with palliative intent, and one with first curative and then palliative intent.

**Table 2:** Overview of treatment for 76 patients with malignant orbital tumors.

Treatment	Primary orbital	Secondary invading	Lymphoproliferative	Metastases	SUM
Radiotherapy only	1	4	4	4	13
Chemotherapy only	0	0	3	1	4
Surgery only^a^	1^c^	5^b^	1^d^	2^e^	9
Radio- and chemotherapy	1	3	5	2	11
Surgery and radiotherapy	4	14	1	0	19
Surgery and chemotherapy	1	0	1	0	2
Surgery, radiotherapy, and chemotherapy	2	9	0	1	12
No treatment	0	0	0	2^f^	2
Unknown	1^g^	0	3^g^	0	4
SUM	11	35	18	12	76

a Surgery does not include incisional biopsies. Radiotherapy includes photon-, proton- and carbon ion radiation, and interstitial brachytherapy.

b Four exenterations and one anterior orbitotomy.

c One partial resection via lateral orbitotomy.

d Lateral orbitotomi at HUH, further treatment at another hospital.

e Two anterior orbitotomies.

f Metastases from prostate cancer and merkel cell carcinoma.

g Only biopsy at HUH, further treatment at other hospitals. Immunotherapy or repeated surgeries are not included.

**Table 3:** Overview of surgical treatment for patients with malignant orbital tumors.

Treatment	Primary orbital	Secondary invading	Lymphoproliferative	Metastases	SUM
Any surgery except biopsies	8	28	3	3	42
Exenteration	3	20	0	0	23
Other surgical treatment:	5	8	3	3	19
Anterior orbitotomy	1	2	1	2	6
Lateral orbitotomy	3	2	2	0	7
Transcranial orbitotomy	0	1	0	0	1
Lateral rhinotomy	0	2	0	0	2
Eviscerasjon of eye with transplant	1	0	0	0	1
Transnasal endoscopic resection^a^	0	1	0	0	1
Extirpation/resection of cranial bone tumor	0	0	0	1	1

a Transnasal endoscopic resection of skull base tumor in Ottawa, followed by endoscopic endonasal anterior skull base chondrosarcoma resection.

**Figure 3:**
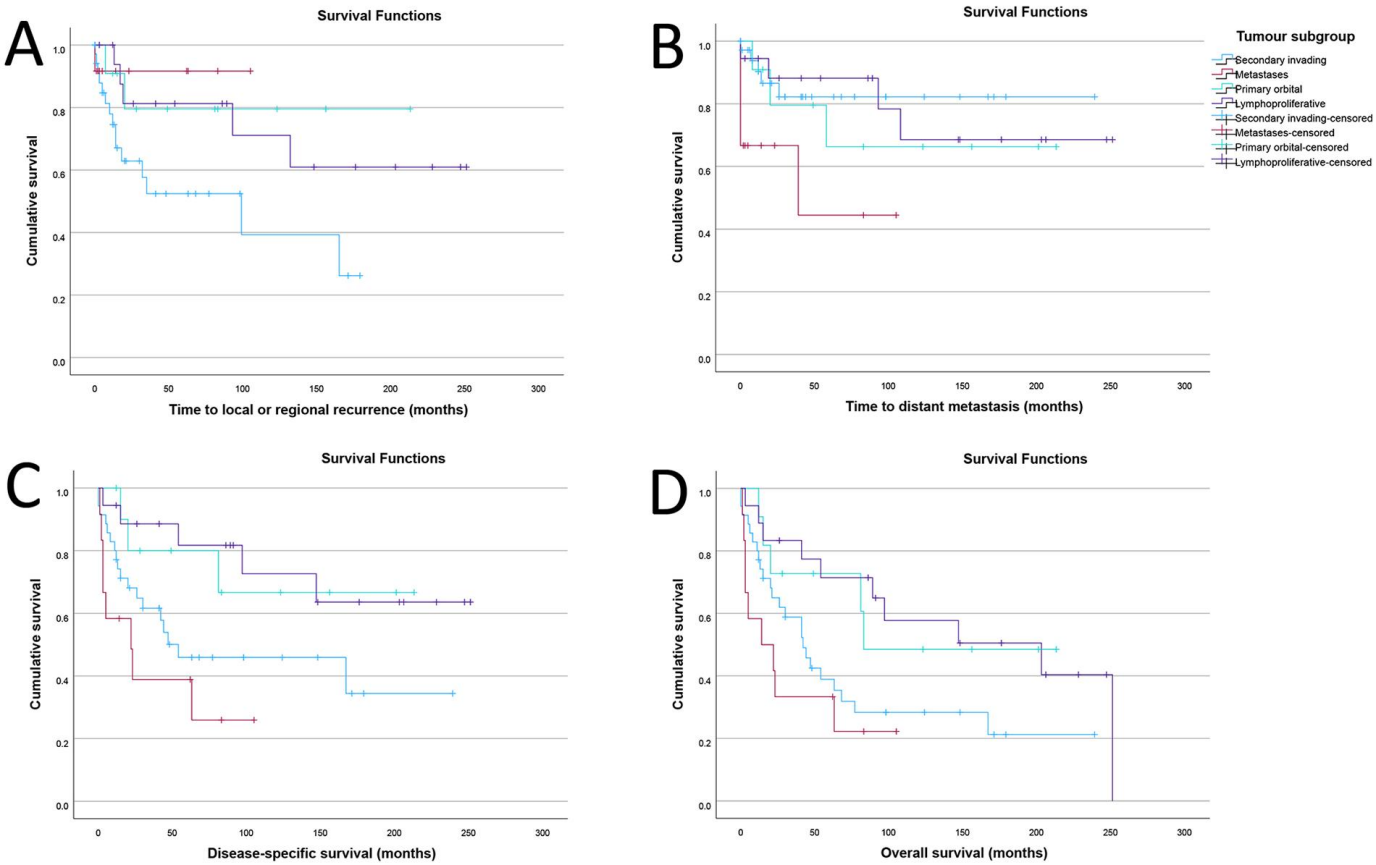
Kaplan–meier plots. **A**: time to local or regional recurrence for 23 patients with orbital tumors (*p* = 0.066, pooled log-rank). **B**: time to distant metastasis for 17 patients with orbital tumors (*p* = 0.052, pooled log-rank). **C**: disease specific survival for patients with orbital tumors including secondary invading orbital tumors (*n* = 35), primary orbital tumors (*n* = 11), lymphoproliferative orbital malignancies (*n* = 18) and orbital metastases (*n* = 12) (*p* = 0.014, pooled log-rank). **D**: overall survival for patients with orbital tumors including secondary invading orbital tumors (*n* = 35), primary orbital tumors (*n* = 11), lymphoproliferative orbital malignancies (*n* = 18) and orbital metastases (*n* = 12) (*p* = 0.026, pooled log-rank).

A total of 32 (42%) patients had one or more local/regional recurrences and/or distant metastases after orbital involvement (Figures 3A,B, and Supplementary Table 1). Median time to the first local recurrence was 15 months (2–165 months), median time to first regional recurrence was 14 months (0–132), and median time to first distant metastasis was 12 months (0–108 months).

Survival-data is presented in Figure 3 as Kaplan-Meyer plots, and in Supplementary Table 2. Out of 76 patients, 48 (63%) were dead by the end of the study. Five-year OS was 51% and 5-year DSS was 59%, with significant variations between subgroups. Lymphoproliferative diseases had significantly longer OS and DSS compared to metastases (*p* = 0.008, *p* = 0.003, respectively, pairwise log-rank) and secondary invasive tumors (*p* = 0.041, *p* = 0.033, respectively) (Figures 3C,D). Primary orbital tumors had longer DSS than metastases (*p* = 0.033). From the Kaplan Meier plots, primary orbital tumors also appeared to have longer survival than secondary invasive tumors but the difference was not significant (OS: *p* = 0.131, DSS: *p* = 0.136).

Median OS was 63 months [95% confidence interval (CI) 28–98 months] and median DSS was 147 months (95% CI 30–264 months). Median time to death by any cause was 21 months (0–251 months) and median time to tumor-related death was 15 months (0–167 months). Median follow-up was 46 months (0–251 months).

## Discussion

This comprehensive overview, spanning a 22-year period, provides outcome data for patients with malignant orbital tumors. Secondary invading tumors were the most frequent, followed by lymphoproliferative, metastatic, and primary orbital lesions, consistent with findings from other Western populations. Disease-specific five-year survival varied markedly between subgroups, with the most favourable outcomes seen in lymphoproliferative and primary orbital tumors and the poorest in secondary invading tumors and orbital metastases. Despite the small patient volume, the overall results, including treatment patterns and survival rates, were comparable to those reported in larger international series.

Generally, malignant tumors correspond to approximately 1/3 of all tumors in the orbit (2–6), although in some studies the occurrence has been higher (45%–63%) (1, 7, 8). This could be explained by the inconsistent definitions of orbital tumors and the anatomical limits of the orbit in previous literature. Some studies have included intraocular tumors (8) while others have required invasion of Tenon's capsule for the tumor to be included (7). For tumors adjacent to the orbit, some include tumors without bony erosion while others require bony erosion as evidence of orbital invasion (39). There are also differences in subcategorization of orbital tumors, where some studies include lymphoproliferative disorders as primary orbital (2, 7, 8), and some include benign tumors as secondary invading (4, 5, 40).

Although we observed some variation in tumor subtypes, the yearly incidence of malignant orbital tumors was relatively constant in the population from Western Norway with 0,30 tumors per 100 000 inhabitants in this study compared to 0,32 tumors per 100 000 inhabitants in the period from 1961 to 1999 (3).

The proportion of malignant tumors with primary orbital origin was 14% in our material. Similar studies have reported a proportion of 43%–66% (2, 7, 8) but this includes lymphoproliferative diseases and/or ocular tumors. By including lymphoproliferative diseases in this category, we reach a comparable proportion of 38%. The lacrimal gland represented the most frequent site of origin which is in agreement with findings from earlier studies (8, 15, 41).

The proportion of secondary invading tumors (46%) is consistent with comparable studies where secondary invading tumors represent 25%–48% of all orbital malignancies (2, 7, 42), and that most invading carcinomas originated in the paranasal sinuses (39). We found a lower frequency of invasive choroidal or conjunctival malignant melanomas (4, 5, 7), and invasive carcinomas from eyelids (2, 40, 42) which may reflect a reduced risk in our cohort for invasive disease after primary treatment of these patients.

Non-Hodgkin lymphomas accounted for 24% of all malignant orbital tumors in our series consistent with previous reports identifying lymphomas as among the most common orbital malignancies, with proportions ranging from 11% to 26% (1–4, 7). The relatively higher incidence of follicular lymphomas is most likely explained by the limited number of patients (10, 43, 44).

Metastases represented 16% of all malignant orbital tumors among our patients, which is slightly lower compared to our previous study where metastases were seen in 22% of the patients (3). Other investigators have reported metastases as 1%–13% of all orbital tumors (2, 4, 5, 7, 40, 42, 45–49).

For malignant tumors originating in the lacrimal gland, the 5-year OS in our cohort was 57%. This is comparable to the 59% 5-year OS reported by Rose and co-workers (50) in a large series of lacrimal gland carcinomas. Similarly, Bonavolonta and co-workers (41) observed 5-year OS rates of 52% and 37%, following eye-sparing surgery and exenteration, respectively.

Although the prognosis is generally poor for patients diagnosed with secondary invading tumors, this depends on histology, tumor size and degree of invasion (39, 51–60). We observed a 5-year DSS of 46%, despite that almost 1/3 of the patients were presenting with a relapse of a former extra-orbital tumor and that nearly all patients in our study were stage T4. For sinonasal tumors, invasion into the orbital apex is a poor prognostic sign where surgery is generally not advocated (12, 56). In contrast, sinonasal tumors only extending to the perioorbita and not involving the soft tissues of the orbit can be managed without exenteration in combination with radiotherapy (12, 14, 61). Our outcome data with a 5-year DSS and OS of 46% and 39% respectively is comparable to other studies reporting a 5-year DSS of 44% (54, 59). The large proportion of secondary invading tumors explains the relatively high frequency of exenterations.

In our cohort, the 5-year DSS for orbital lymphoproliferative diseases was 82% and the 5-year OS was 71%. In a review, Hassan and co-workers reported a comparable 5-year OS of 75.9% (62) while Vest and co-workers found a 5-year DSS of 87.5% and a 5-year OS of 73.8% for lymphomas of the lacrimal gland (44). Variations in the distribution and biological behavior of non-Hodgkin lymphoma subtypes likely account for some of the differences observed between studies.

The median time from diagnosis of the primary tumor to metastasis in the orbit was 43 months. This is within the range reported in other studies of 40–52 months (45, 46). Orbital metastases are associated with poor prognosis as they reflect extensive disease. In our material, the median DSS was 22 months, but half of the patients who died of tumor-related causes, died within 4 months of diagnosis. We found a relatively high 5-year DSS of 39%, compared to Magliozzi and co-workers who reported 14% 2-year survival (45) and Valenzuela and co-workers who reported 29% alive after 17 months (48). This could be due to the relatively high proportion of patients having metastases from carcinoids that had long survival times (63).

For most conditions that require complex, procedural care, patient outcome in high-volume institutions is better than in low-volume institutions (64). The strength of the relationship may vary, however, depending among others on the complexity of the procedures and a variety of hospital-related factors. No studies have examined volume-outcome relationships for orbital malignancies. For head and neck malignancies including sinonasal and skull base tumors, high volume contributes to improved outcome (65). Interestingly, Eskander and co-workers observed that for these malignancies, hospital related factors seemed to be more important than those of the surgeon (66). We have structured our clinical practice such that patients with orbital malignancies are managed by a multidisciplinary team with broad clinical experience in related conditions addressing the challenges posed by the rarity of these tumors.

### Limitations

Its retrospective design and case selection based on diagnostic/procedure codes introduce potential selection bias. Patients were included over two decades, during which referral patterns and management strategies evolved. Medical records were sometimes incomplete due to care being provided at other institutions and older, less detailed documentation. The sample size is relatively small, and histologically distinct tumors were grouped into four heterogeneous categories, so survival estimates are likely influenced by the distribution of subtypes with differing chemo- and radio sensitivity.

## Conclusion

Orbital malignancies represent a wide range of histological diagnoses with differences in presenting signs, therapeutic options, and prognosis. The observed outcome from HUH in terms of DSS and OS as well as recurrences are within the range reported in the international literature. Further examination of volume-outcome relationships in orbital disorders should be performed.

## Data Availability

The original contributions presented in the study are included in the article/supplementary materials, further inquiries can be directed to the corresponding author.
