# Supplementary material for: Outcomes of malignant orbital tumors: a 22-year Norwegian single-center series
Source: Front Surg. 2026 Jun 24;13:1862307. doi: 10.3389/fsurg.2026.1862307 (PMC13341615; doi:10.3389/fsurg.2026.1862307)
Supplement: Supplementary file 1 [file Datasheet1.pdf]

Supplemental information

Identification of patients

The patients were identified by searching for relevant codes in the Hospital's database from January 1999 to December 2020. We included 1: Procedure codes relevant for surgical treatment of orbital tumours; CAA30 (biopsy of orbit), CAB00 (anterior orbitotomy with excision of lesion), CAB10 (lateral orbitotomy with excision of lesion), CAB40 (transcranial orbitotomy with excision of lesion), and CAD00 (orbital exenteration with use of graft or flap), 2: Diagnostic codes C69.5 (malignant neoplasm of lacrimal gland or duct), C69.6 (malignant neoplasm of orbit) and C69.8 (malignant neoplasm: overlapping lesion of eye and adnexa), 3: Diagnostic codes C41.0 (malignant neoplasm of bones of skull and face), diagnostic codes starting with C81-C96 (different lymphomas), and diagnostic codes starting at C31 (malignant neoplasm of accessory sinuses) limited to patients that had been examined at the Department of Ophthalmology, and 4: Diagnostic codes C44.1 (malignant neoplasm of skin of eyelid) and C44.3 (malignant neoplasm of skin of other or unspecified parts of face) that had received radiotherapy and had been examined at the Department of Ophthalmology.

Definitions

Different definitions of the orbit exist in the scientific literature. In this study the orbit is defined as the tissue posterior to the orbital septum and limited by the orbital walls. This includes extraocular muscles, lacrimal gland, fat, vessels, and nerves, and excludes eyelids, eyeball, lacrimal sac, and conjunctiva.

Time of diagnosis was defined as the date of histologically confirmed diagnosis. Duration of symptoms was defined as time from start of symptoms to histologically confirmed diagnosis. Proptosis was defined as a protruding eye with a difference of ≥ 2 mm compared to the unaffected eye measured with a Hertel exophthalmometer. Elevated pressure was defined as intraocular pressure ≥ 21mmHg or a difference of ≥

5mmHg compared to the unaffected eye measured with iCare or Goldmann Applanation Tonometry. Reduced visual acuity was defined as visual acuity ≤ 0.63.

The main endpoints were overall survival (OS), disease specific survival (DSS), and time to recurrence. Overall survival was defined as time from diagnosis to death of any cause, or date of last contact, with the latter being a censored event. Disease specific survival was defined as time from diagnosis to death of tumour-related cause, or date of last contact or death of any other cause, with the two latter being censored events. Time to recurrence was defined as time from diagnosis to local recurrence, regional recurrence or distant metastases, or date of last contact or death of any cause, with the two latter being censored events. If extra-orbital metastases were present at time of diagnosis, time to recurrence was defined as 0 months. Median OS was defined as time from diagnosis to 50% of the patients died of any cause. Median DSS was defined as time from diagnosis to 50% of the patients died from tumour-related causes.

Radiation therapy

Radiation therapy included carbon-ion radiotherapy in three patients (two at HIT, Heidelberg, Germany, one at CNAO, Pavia, Italy), proton beam therapy (one in Heidelberg) and interstitial brachytherapy (one in Heidelberg). The rest of the patients received megavoltage photon radiotherapy. Since 2005 our institution has used intensity-modulated radiation therapy (IMRT) and later Volumetric Modulated Arc Therapy (VMAT) for these patients. The radiation dose varied between 8 - 75 Gy, with a median dose of 64 Gy, most often in fractions of 2 Gy/day (1.5 - 8 Gy).

Supplemental tables

Supplemental table 1. Staging, recurrences and supplementary survival data for different categories of malignant orbital tumours

	Primary	Secondary	Lymphoproliferative	Metastases
**Number of patients**	11	35	18	12
**Median duration of symptoms (range) months**	6 (0-24)	3 (0-36)	4 (0-17)	3 (0-9)
**Stage**				
**TNM, n**				
**T2**	4	0		NA
**T3**	2	7		NA
**T4**	3	22		NA
**Unclassified**	2[1]	6[2]		
**Ann Arbor, n**				
**IE**			9	
**III**			1	
**IV**			5	
**Unclassified**			3[3]	
**Recurrences n**	4	17	6	5
**local n**	1	12	3	0

**regional n**	1	6	3	1
**metastasis n**	3	5	4	5
**Median time to recurrence (range) months**				
**local or regional**	14 (7-20)	12 (0-165)	19 (13-132)	0
**metastasis**	20 (8-58)	12 (0-26)	56 (0-108)	0 (0-39)
**Deceased (tumour-related cause) n**	5 (3)	24 (18)	10 (5)	9 (8)[4]
**Median DSS (95% CI) months**	NA[5]	54 (0-151)	NA[5]	22 (0-49)
**Median OS (95% CI) months**	NA[5]	42 (24-60)	203 (39-367)	14 (0-43)
**Median time to death (range) months**				
**tumor-related**	20 (15-81)	14 (0-167)	54 (3-147)	4 (1-63)
**any cause**	20 (12-83)	24 (0-167)	72 (3-251)	5 (1-63)

[1] 1 lacrimal gland tumour, 1 extramedullary myeloid carcinoma
[2] 4 lacrimal sac tumours, 1 malignant odontogenic myxoma, 1 sphenoidal chondrosarcoma
[3] 1 follicular lymphoma, 1 plasma cell myeloma, 1 B-cell lymphoma
[4] The three patients alive at the end of the study had metastases from ileal carcinoid, colonic carcinoid, and malignant melanoma.
[5] Not available (NA) - mortality never reached 50%

Supplemental table 2: Overview of survival for different groups of orbital malignancies.

**Survival**	**Primary orbital**		**Secondary invading**		**Lymphoproliferative**		**Metastases**		**Total**	
	**DSS**	**OS**	**DSS**	**OS**	**DSS**	**OS**	**DSS**	**OS**	**DSS**	**OS**
1-year	100%	100%	80%	80%	94%	94%	58%	58%	83%	83%
3-year	80%	73%	62%	59%	89%	83%	39%	33%	67%	63%
5-year	80%	73%	46%	39%	82%	71%	39%	33%	59%	51%
Median follow-up, months (range)	81 (12-213)		41 (0-239)		94 (3-251)		18 (1-105)		46 (0-251)	

DSS, Disease Specific Survival; OS, Overall Survival.
